# Regional Variation of CH_4_ and N_2_ Production Processes in the Deep Aquifers of an Accretionary Prism

**DOI:** 10.1264/jsme2.ME16091

**Published:** 2016-09-03

**Authors:** Makoto Matsushita, Shugo Ishikawa, Kazushige Nagai, Yuichiro Hirata, Kunio Ozawa, Satoshi Mitsunobu, Hiroyuki Kimura

**Affiliations:** 1 Department of Environment and Energy Systems, Graduate School of Science and Technology, Shizuoka UniversityOya, Suruga-ku, Shizuoka 422–8529Japan; 2 Department of Geosciences, Faculties of Science, Shizuoka UniversityOya, Suruga-ku, Shizuoka 422–8529Japan; 3 Center for Integrated Research and Education of Natural Hazards, Shizuoka UniversityOya, Suruga-ku, Shizuoka 422–8529Japan; 4 Department of Environmental Conservation, Graduate School of Agriculture, Ehime UniversityTarumi, Matsuyama 790–8566Japan; 5 Research Institute of Green Science and Technology, Shizuoka UniversityOya, Suruga-ku, Shizuoka 422–8529Japan

**Keywords:** accretionary prism, deep aquifer, methanogens, fermentative bacteria, denitrification

## Abstract

Accretionary prisms are mainly composed of ancient marine sediment scraped from the subducting oceanic plate at a convergent plate boundary. Large amounts of anaerobic groundwater and natural gas, mainly methane (CH_4_) and nitrogen gas (N_2_), are present in the deep aquifers associated with an accretionary prism; however, the origins of these gases are poorly understood. We herein revealed regional variations in CH_4_ and N_2_ production processes in deep aquifers in the accretionary prism in Southwest Japan, known as the Shimanto Belt. Stable carbon isotopic and microbiological analyses suggested that CH_4_ is produced through the non-biological thermal decomposition of organic matter in the deep aquifers in the coastal area near the convergent plate boundary, whereas a syntrophic consortium of hydrogen (H_2_)-producing fermentative bacteria and H_2_-utilizing methanogens contributes to the significant production of CH_4_ observed in deep aquifers in midland and mountainous areas associated with the accretionary prism. Our results also demonstrated that N_2_ production through the anaerobic oxidation of organic matter by denitrifying bacteria is particularly prevalent in deep aquifers in mountainous areas in which groundwater is affected by rainfall.

Sequences in the accretionary prism of the non-subducting continental crust are thick sediments that were originally deposited on the subducting ocean plate. During subduction, parts of the marine sediment on the ocean plate are scraped off and accreted into an accretionary prism in the overlying continental plate (56). Accretionary prisms have been reported at convergent plate boundaries around the world, including those at Alaska and Washington in the U.S., New Zealand, Chile, Peru, Indonesia, Taiwan, and Japan (13, 21, 27).

The accretionary prism in southwest Japan, known as the Shimanto Belt, is composed of marine sediments deposited on the Philippine Sea Plate during the Cretaceous and Tertiary Periods (27, 55). The sediment structure is currently distributed in a wide region from the coastal area of the Pacific Ocean side to the mountainous area, and is traceable for 1,800 km in southwest Japan (Fig. 1). This accretionary prism is derived from ancient marine sediment scraped from the subducting ocean plate, and, thus, is rich in complex organic compounds (28). This sediment contains layers of water-bearing permeable sandstone and water-impermeable shale. Groundwater in this region is recharged by rainfall and seawater, which infiltrates outcrops or faults and is anaerobically reserved in deep aquifers (29). In addition to anaerobic groundwater, a large amount of natural gas, mainly methane (CH_4_), has been detected in deep aquifers (29, 47).

CH_4_ is an important greenhouse gas and energy resource generated predominantly by methanogenic archaea and through the thermal degradation of organic molecules in sediments. A previous study indicated that the anaerobic biodegradation of organic compounds by hydrogen (H_2_)-producing fermentative bacteria and H_2_-utilizing methanogens contributes to the significant CH_4_ reserves in deep aquifers associated with the accretionary prism in southwest Japan (20, 26, 29). However, regional variations in the CH_4_ production process are poorly understood, in part because of insufficient data. In addition to CH_4_, a large amount of nitrogen gas (N_2_) is present in natural gas derived from deep aquifers. The origin of this N_2_ remains elusive.

Therefore, the objectives of this study were to identify the origin of CH_4_ reserved in deep aquifers in the Shimanto Belt by analyzing the stable isotopic signatures of groundwater and natural gas samples. We also examined the processes of and potential for microbial CH_4_ and N_2_ production using culture experiments and a DNA analysis. Collectively, the results from geochemical analyses and microbiological experiments were used to develop a model that explains regional variations in microbial activities and geochemical cycles in deep aquifers associated with the accretionary prism in southwest Japan.

## Materials and Methods

### Study sites and sample collection

**Fig. 1:**
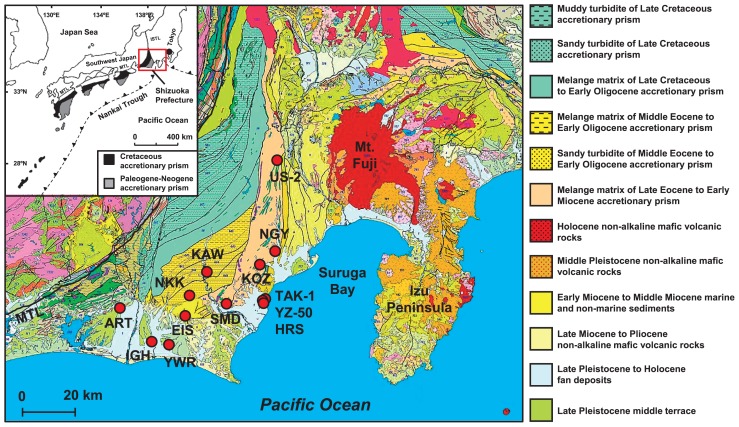
Geological map of the study area. The location of the accretionary prism known as the Shimanto Belt in southwest Japan is according to Kano *et al.* (27). The geological map of Shizuoka Prefecture, Japan, is modified from the 1:200,000 seamless digital geological map of Japan (14). The circles indicate the locations of the wells used for sampling. MTL, Median Tectonic Line; ISTL, Itoigawa-Shizuoka Tectonic Line.

Groundwater and natural gas samples were collected from 13 wells situated in Shizuoka Prefecture, Japan (Fig. 1). The wells were drilled down to deep aquifers associated with the accretionary prism and constructed from tight steel-casing pipes including strainers (Table S1). Groundwater flows into these wells through parts of the strainers. Groundwater rises up to ground level by natural water pressure or is anaerobically drawn up to ground level by a water pump or gas lift system.

In order to prevent contamination by air and water from shallow environments, groundwater was pumped for 24 h before sampling. Groundwater samples were collected under anaerobic conditions into autoclaved serum bottles using a sterile silicone tube. The concentrations of dissolved natural gas were so high that gas exsolved at ground level. Natural gas samples were collected in an inverted funnel underwater and then directed into autoclaved serum bottles. Serum bottles were tightly sealed underwater with sterile butyl-rubber stoppers and aluminum crimps to prevent contamination by air.

### Physical and chemical parameter measurements

The physical and chemical parameters of groundwater were measured at the outflow of the wells. Temperature was measured with a CT-460WR thermometer (Custom, Tokyo, Japan). Oxidation-reduction potential (ORP) and pH were measured with RM-20P and HM-20P portable meters (DKK-TOA, Tokyo, Japan), respectively. Electric conductivity (EC) was measured with a CM-21P portable meter (DKK-TOA).

The concentrations of anions (HCO_3_^−^, CI^−^, Br^−^, I^−^, F^−^, NO_2_^−^, PO_4_^2−^, NO_3_^−^, SO_4_^2−^, acetate, and formate) and cations (Na^+^, K^+^, Mg^2+^, Ca^2+^, and NH_4_^+^) were measured using an ICS-1500 ion chromatography system (Dionex, Sunnyvale, CA, USA). Sulfide was analyzed using a methylene blue method (8). Total iron was measured using a modified version of the ferrozine method (54). Ferrous iron was quantified using a modified version of the 1,10-phenanthroline method (58). Spectrophotometric analyses for sulfide and irons were performed on site with a DR/2400 portable spectrophotometer (Hach, Loveland, CO, USA). Dissolved organic carbon (DOC) in groundwater filtered through pre-combusted GF/F glass microfiber filters (Whatman, Maidstone, UK, USA) was measured with a TOC-V total organic carbon analyzer (Shimadzu, Kyoto, Japan).

H_2_, N_2_, O_2_, N_2_O, CO_2_, and CH_4_ concentrations in natural gas were measured with a GC-2014 gas chromatograph (GC) (Shimadzu) equipped with a thermal conductivity detector (TCD) and packed column (ShinCarbon ST, 6.0 m×3.0 mm i.d.; Shinwa Chemical Industries, Kyoto, Japan). The GC conditions used were as follows: injector temperature, 170°C; column oven temperature, 150°C; detector temperature, 170°C. Argon (Ar) was used as a carrier gas at a constant flow mode of 50 mL min^−1^. N_2_ and Ar concentrations were measured with GC-2014 GC (Shimadzu) equipped with TCD and a packed column (Molecular Sieve 5A, 3.0 m×3.0 mm i.d.; Shinwa Chemical Industries). The GC conditions used were as follows: injector temperature, 50°C; column oven temperature, 40°C; detector temperature, 50°C. Helium was used as a carrier gas at a constant flow rate of 50 mL min^−1^. CH_4_, C_2_H_6_, and C_3_H_8_ concentrations were measured with GC-2014 GC (Shimadzu) equipped with a flame ionization detector and packed column (Sunpak-A, 2.0 m×3.0 mm i.d.; Shinwa Chemical Industries). The GC conditions used were as follows: injector temperature, 100°C; column oven temperature, 65°C; detector temperature, 100°C. N_2_ was used as a carrier gas at a constant flow rate of 50 mL min^−1^. Samples were analyzed in triplicate. Reference gases were analyzed at the start of each gas analysis. The confidence limits of the measurement were 0.01 vol.% for H_2_, N_2_, O_2_, N_2_O, Ar, CO_2_, and CH_4_, and 0.001 vol.% for C_2_H_6_ and C_3_H_8_.

### Analysis of the stable carbon isotopic ratio

The stable carbon isotope ratio (^13^C/^12^C) of CH_4_ in natural gas was measured with a Trace GC Ultra gas chromatograph (Thermo Fisher Scientific, Waltham, MA, USA) that was connected to a Delta V Advantage isotope ratio mass spectrometer (IRMS) with a GC IsoLink conversion unit and ConFlo IV interface (Thermo Fisher Scientific) (6).

The ^13^C/^12^C of total dissolved inorganic carbon (∑CO_2_) in groundwater, mainly bicarbonate, was analyzed as described previously (36). Groundwater samples for analyzing the ^13^C/^12^C of ∑CO_2_ were fixed with 0.5 mL of saturated HgCl_2_ solution and sealed with sterile butyl-rubber stoppers and aluminum crimps with no air bubbles. A 10-mL headspace was created inside each serum bottle with pure helium gas and acidified by adding CO_2_-free H_3_PO_4_ solution. Sample bottles were left in the dark for 24 h to achieve equilibrium between dissolved CO_2_ and headspace CO_2_. CO_2_ in this headspace was subsampled, and the ^13^C/^12^C ratio of CO_2_ was measured by a Trace GC Ultra gas chromatograph (Thermo Fisher Scientific) that was connected to a Delta^plus^ XL IRMS (Thermo Fisher Scientific).

The stable isotope ratio was expressed in the conventional δ notation calculated from the equation

$$\delta =[R_{\text{sample}}/R_{\text{standard}}-1]\times 1000\;[‰],$$

where *R* is the isotope ratio (^13^C/^12^C). The isotope ratio in this study is reported relative to the international standard, Vienna Pee Dee Belemnite (VPDB). The standard deviations of the δ^13^C of CH_4_ and CO_2_ were ±0.3‰ and ±1‰, respectively.

### Total cell count and catalyzed reporter deposition fluorescence *in situ* hybridization (CARD-FISH)

The groundwater samples used for the total cell count were fixed in neutralized formalin (final concentration 1%). Ten milliliters of a groundwater sample was filtered using pre-blackened polycarbonate filters (pore size, 0.2 μm; diameter, 25 mm) (Millipore, Billerica, MA, USA). Microbial cells collected on the filter were stained with SYBR Green I (1:100 dilution) (Life Technologies, Carlsbad, CA, USA) (39). Microbial cells were observed under a model BX51 epifluorescence microscope equipped with a U-MNIB3 fluorescence filter (Olympus, Tokyo, Japan), and more than 50 microscopic fields (average 20–30 cells in each field) were counted for each sample. Cell counting was performed within 24 h of groundwater sampling.

Regarding CARD-FISH targeting archaeal and bacterial 16S rRNAs, groundwater samples were collected from eight wells (KAW, YZ-50, NKK, EIS, SMD, KOZ, US-2, and ART). CARD-FISH was conducted following the protocols reported by Mitsunobu *et al.* (35). Briefly, 50 mL of each groundwater sample was filtered with white polycarbonate membrane filters (pore size, 0.2 μm; diameter, 25 mm; Advantec, Tokyo, Japan). Cells on the filters were fixed in 3% paraformaldehyde and dehydrated in ethanol. The cells were then hybridized using the following horseradish peroxidase-labeled probes: *Bacteria*-specific EUB338 (2), *Archaea*-specific ARCH915 (52), and the control probe Non338 (60). In order to overcome the high autofluorescence of clay particles, cells were counterstained with SYBR Green I (Life Technologies). The Cy3-labeled tyramide signal was amplified using a TSA-Plus cyanine 3 system (Perkin Elmer, Waltham, MA, USA). Cell counting was performed with a model BX51 epifluorescence microscope (Olympus) equipped with a U-MNIG3 filter (Olympus) for hybridized cells and the U-MNIB3 filter (Olympus) for SYBR Green I-stained cells.

### Next generation sequencer (NGS) analysis of 16S rRNA genes

In the NGS analysis, eight groundwater samples were collected from the same wells as those used in the CARD-FISH analysis. Ten liters of groundwater samples was aseptically filtered with Sterivex-GV filter units (pore size, 0.22 μm; Millipore) using a sterilized silicone tube and tubing pump (51). The bulk DNAs of microorganisms trapped by the filter units were extracted using a MORA-EXTRACT kit (Kyokuto Pharmaceutical, Tokyo, Japan). Bacterial and archaeal 16S rRNA gene fragments were simultaneously amplified from bulk DNA by PCR using the primer set, Pro341f and Pro806r based on the V3–V4 hypervariable region of the prokaryotic 16S rRNA gene (57). Library generation and sequencing using an Illumina Miseq sequencer were performed according to the method described by Takahashi *et al.* (57). Analyses of sequence reads were performed using the Ribosomal Database Project (RDP) Classifier Version 2.10 with a confidence threshold of 80% (61). The relative abundance of each phylogenetic group was assessed by the number of sequence reads affiliated with that group. In order to better estimate relative abundance, the number of sequence reads affiliated with that group was adjusted based on the mean 16S rRNA gene copy numbers for that group provided by the rrnDB database (53).

In order to assess microbial diversity, sequence reads were grouped into operational taxonomic units (OTUs) sharing more than 97% sequence similarity, and alpha-diversity indices (Chao 1 and Shannon index) and coverage percentages were then calculated using the Quantitative Insights Into Microbial Ecology (QIIME) v 1.5.0 pipeline (7).

### Measurements of potential microbial gas production

Autoclaved 70-mL serum bottles were tightly sealed with sterile butyl-rubber stoppers and aluminum crimps. Serum bottles were vacuum-pumped and then filled with pure N_2_. After this process had been repeated four times, the serum bottles were vacuum-pumped again. Thirty milliliters of each groundwater sample was anaerobically injected into serum bottles with 35-mL syringes and needles.

In order to assess the potential for CH_4_ production by methanogenic archaea, we prepared enrichments using groundwater amended with methanogenic substrates. Groundwater was supplemented with acetate (20 mM), methanol (20 mM), formate (20 mM), or H_2_/CO_2_ (80:20, v/v; 150 kPa). Except for H_2_/CO_2_-supplemented bottles, the headspaces of serum bottles were filled with pure N_2_ at 150 kPa. Enrichments using groundwater amended with organic substrates were also prepared in order to evaluate the potential for microbial H_2_ production via the anaerobic biodegradation of organic matter. Groundwater samples were amended with 3 mL of yeast extract, peptone, and glucose (YPG) medium (10 g of yeast extract, 10 g of peptone, and 2 g glucose L^−1^ distilled water) and 20 mM of 2-bromoethanesulfonate (BES), an inhibitor of methanogens (29). The headspaces of the serum bottles were filled with pure N_2_ at 150 kPa. We also measured the potential for CH_4_ production by the syntrophic biodegradation of fermentative bacteria and methanogenic archaea. Groundwater samples were supplemented with 3 mL of YPG medium. In these enrichments, BES was not added. The headspaces of the serum bottles were filled with pure N_2_ at 150 kPa.

The potential for N_2_ production by denitrifying bacteria was also assessed. Groundwater samples were supplemented with 3 mL of YPG medium and nitrite (10 mM) or nitrate (10 mM). The headspaces of the serum bottles were filled with pure CH_4_ at 150 kPa.

These enrichments were anaerobically incubated without shaking at each temperature of groundwater sample that was measured at the outflow of the well. Additionally, enrichments were incubated at temperatures that were 10°C higher than those of groundwater because the actual temperature in a deep aquifer is generally considered to be higher than that of groundwater measured at the outflow of the well (33, 43). H_2_, N_2_, N_2_O, CH_4_, and CO_2_ concentrations in the headspace were measured with GC-2014 GC (Shimadzu) equipped with TCD and a ShinCarbon ST packed column (Shinwa Chemical Industries) as described above. Enrichments were performed in triplicate.

Microorganisms that grew in the enrichments in which biogas production was observed were identified according to the 16S rRNA gene clone library method described in a previous study (29). Briefly, cells in the enrichments were collected by centrifugation and bulk DNA was extracted. Archaeal and bacterial 16S rRNA gene fragments were amplified by PCR from bulk DNA using the *Archaea*-specific primer set, 109aF and 915aR (16, 52), and the *Bacteria*-specific primer set, 8bF and 1512uR (11), respectively. The sequences of the inserted PCR products selected from recombinant colonies were elucidated with an Applied Biosystems 3730xl DNA Analyzer (Life Technologies). The OTUs for each clone library were obtained using GENETYX-Mac ver. 17.0 (Genetyx, Tokyo, Japan). A 3% distance level between sequences was considered the cut-off for distinguishing distinct OTUs. We identified the nearest relative of each OTU using the BLAST program (1). Neighbor-joining phylogenetic trees based on Kimura’s two-parameter model were constructed using the CLUSTAL X version 2.1 program and NJplot software (30, 31, 41, 44). The tree topology was evaluated by bootstrap resampling with 1,000 replicates.

### Nucleotide sequence accession numbers

The 16S rRNA gene sequences obtained in this study have been deposited under GenBank/ENA/DDBJ accession numbers AB848725 to AB848733, AB985755 to AB985758, and DRA004556.

## Results

### Chemical and stable isotopic signatures of groundwater and natural gas

The temperature of groundwater was measured at the outflow of the deep well, and found to range between 24.2°C and 49.3°C (Table 1). pH was between 7.6 and 9.3. The ORP of groundwater indicated the anoxic conditions of deep aquifers associated with this accretionary prism. The EC, an indicator of salinity, varied between 110 mS m^−1^ and 3,090 mS m^−1^. Fe^2+^, Fe^3+^, PO_4_^2−^, NO_2_^−^, NO_3_^−^, SO_4_^2−^, S^2−^, acetate, and formate concentrations in groundwater were low or below the detection limits (Table S2).

In most of the natural gas samples obtained in this study, CH_4_ was the predominant component, accounting for more than 96 vol.% (Table 1). Natural gas sampled from KAW, US-2, ART, and IGH included a large amount of N_2_ (15–50 vol.%) as well as CH_4_. Ar was detected in all natural gas samples (0.06–0.66 vol.%). The ratio of N_2_ to Ar (N_2_/Ar) was between 6 and 203. H_2_, O_2_, N_2_O, CO_2_, and C_3_H_8_ were below the detection limits.

**Fig. 2:**
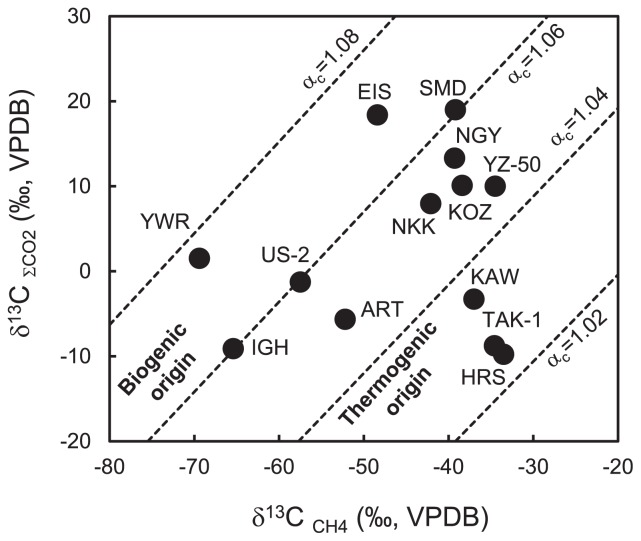
Stable carbon isotope compositions of CH_4_ in natural gas and dissolved inorganic carbon in groundwater. The broken lines of equal carbon isotopic fractionation, α_c_=(δ^13^C_∑CO2_+10^3^)/(δ^13^C_CH4_+10^3^), are drawn for α_c_=1.02, 1.04, 1.06, and 1.08. The categorization of CH_4_ origins is according to Smith and Pallasser (50). VPDB, Vienna Pee Dee Belemnite.

The stable carbon isotope ratios of CH_4_ in natural gas (δ^13^C_CH4_) and dissolved inorganic carbon in groundwater (δ^13^C_∑CO2_), mainly bicarbonate, ranged between −69.4‰ and −33.5‰ and between −9.76‰ and 19.0‰, respectively (Table S3). Carbon isotope fractionation (α_c_) between δ^13^C_∑CO2_ and δ^13^C_CH4_ was between 1.025 and 1.076. We plotted stable isotopic values on a δ^13^C_∑CO2_ versus δ^13^C_CH4_ diagram according to Smith and Pallasser (50). These values fell across regions of biogenic origin by microbial methanogenesis via CO_2_ reduction, regions of thermogenic origin by abiotic CH_4_ production, and the boundary area between the regions of biogenic origin and thermogenic origin (Fig. 2).

### Abundance and diversity of microbial communities in groundwater

Microbial cell densities in groundwater samples ranged between 3.0×10^3^ and 7.7×10^5^ cells mL^−1^ (Table 1). FISH-positive archaeal cells accounted for 5.5% to 68.0% of all microbial cells (Fig. S1 and Table S4). FISH-positive bacterial cells accounted for 7.1% to 45.2% of all microbial cells. The ratios of FISH-positive bacterial cells to archaeal cells (*Bacteria*/*Archaea*) ranged between 0.1 and 5.7.

**Fig. 3:**
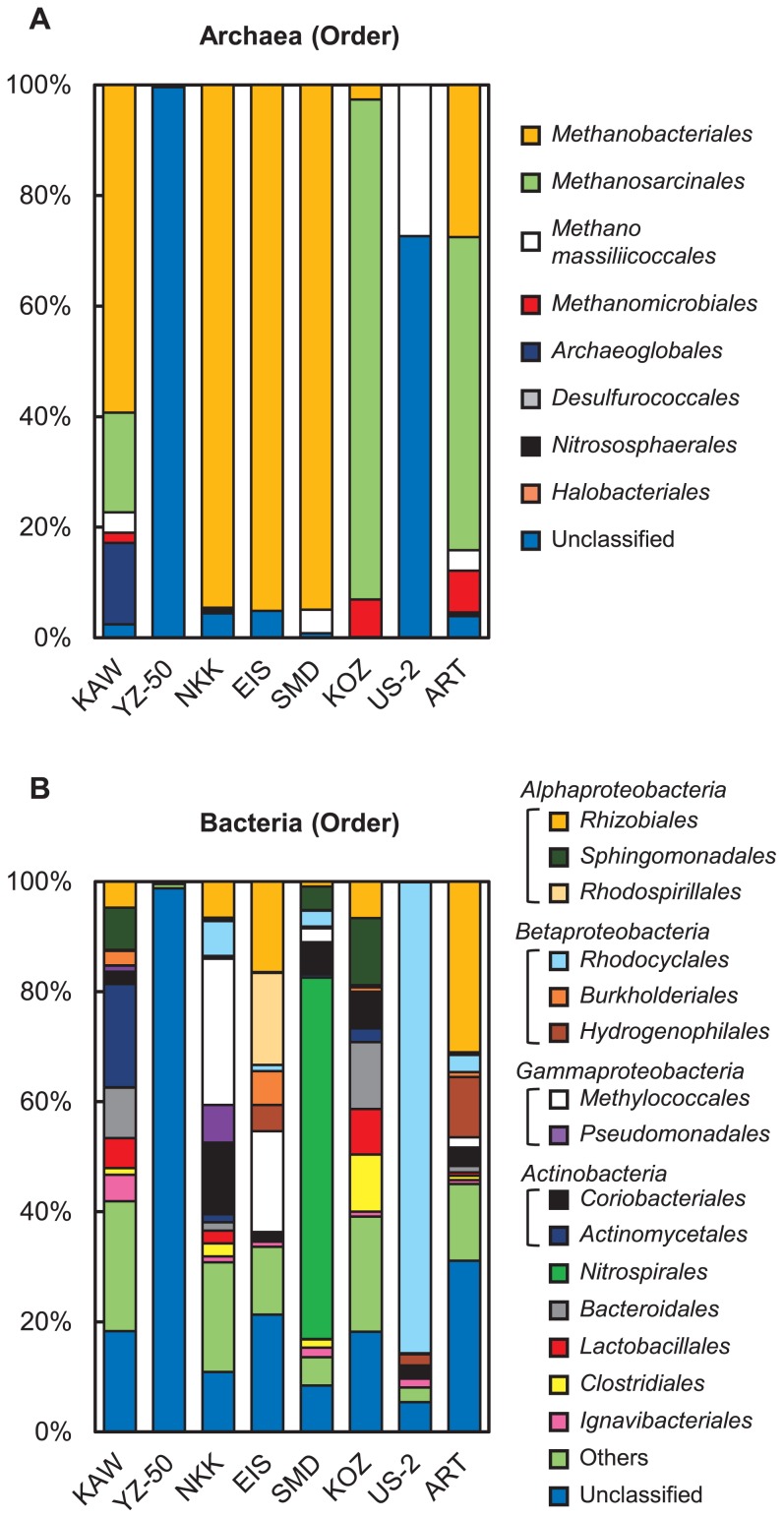
Archaeal and bacterial assemblages in natural groundwater. (A) The relative abundance (%) of archaeal communities. (B) The relative abundance (%) of bacterial communities.

Based on the NGS analysis, 7,787 to 33,274 reads and 128 to 787 OTUs were obtained (Table S5). The coverage percentage reached more than 98%. The Chao1 and Shannon indices ranged between 217 and 1,849 and between 1.18 and 6.25, respectively. The archaeal 16S rRNA genes showed the dominance of methanogens belonging to the order *Methanobacteriales*, an archaeal group that is known to use H_2_ and CO_2_ for growth and methanogenesis, in KAW, NKK, EIS, and SMD (Fig. 3A) (64). We also detected *Methanomassiliicoccales* and *Methanomicrobiales* as minor members of H_2_-utilizing methanogenic archaea (10, 46). On the other hand, the dominance of the order *Methanosarcinales*, which is a methanogenic archaea that uses acetate as a methanogenic substrate, was also indicated in KOZ and ART (25). In contrast, a number of archaeal 16S rRNA genes obtained from YZ-50 and US-2 were unclassified archaea.

The NGS analysis of bacterial 16S rRNA genes demonstrated the dominance of the classes *Alphaproteobacteria*, *Betaproteobacteria*, and *Gammaproteobacteria* (Fig. 3B). The order *Rhizobiales* (*Alphaproteobacteria*), generally known to comprise diazotrophs, was observed in most groundwater samples. We also confirmed the presence of the denitrifying bacterium *Rhodocyclales* (*Betaproteobacteria*) and aerobic methanotrophic bacterium *Methylococcales* (*Gammaproteobacteria*). The class *Actinobacteria* and orders *Nitrospirales*, *Bacteroidales*, *Lactobacillales*, *Clostridiales*, and *Ignavibacteriales*, which are bacterial groups containing anaerobic fermentative bacteria, were also identified (4, 18, 32, 38). A large number of bacterial 16S rRNA genes obtained from YZ-50 were unclassified bacteria.

### Potential for microbial methanogenesis and fermentation

In order to assess the potential for CH_4_ production by methanogens, we anaerobically incubated groundwater samples amended with methanogenic substrates: acetate, methanol, formate, or H_2_/CO_2_. CH_4_ production was only observed in the enrichments amended with H_2_/CO_2_. CH_4_ production was clearly observed in the enrichments using groundwater collected from NKK, SMD, and NGY, and incubations at temperatures that were 10°C higher than those measured at the outflow of the wells exhibited the strong potential for CH_4_ production (Fig. S2 and S3).

We then performed enrichments using groundwater amended with YPG medium and BES in order to assess the potential for H_2_ and CO_2_ production mediated by fermentative bacteria. H_2_ and CO_2_ were detected in the gas phase of bottles within 48 h, except when enrichments using groundwater samples from TAK-1 and HRS were used (Fig. S4 and S5). Incubations at temperatures that were 10°C higher than those of groundwater measured at the outflow of the wells exhibited a stronger potential for H_2_ and CO_2_ production.

**Fig. 4:**
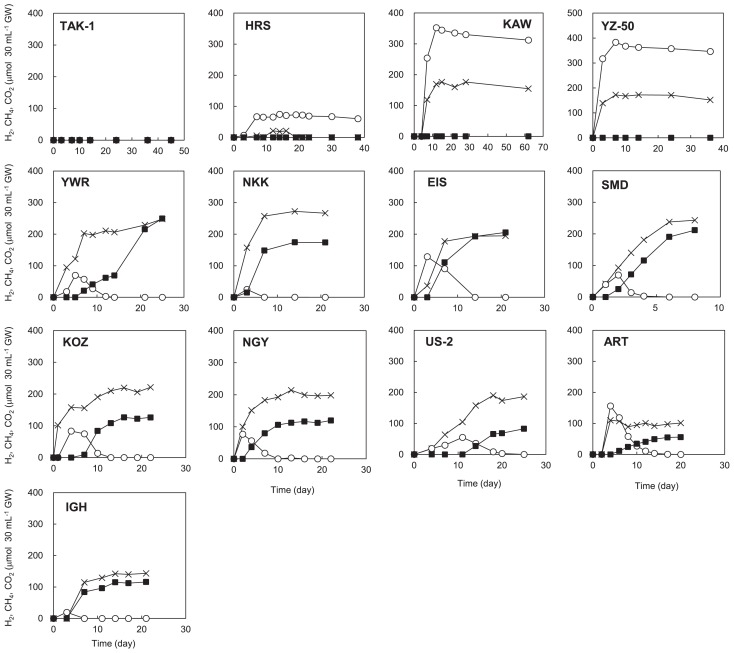
Biogas production from groundwater samples amended with YPG medium incubated at temperatures that were 10°C higher than those of groundwater samples measured at the outflow of deep wells. Data points were obtained from the measurement of cumulative H_2_ (○), CH_4_ (■), and CO_2_ (×) in the gas phase of the culture bottles.

In order to assess whether CH_4_ is produced from organic substrates by the cooperative catabolism of H_2_-producing fermentative bacteria and H_2_-utilizing methanogens, we performed enrichments using groundwater supplemented with YPG medium. CH_4_ production was not observed in the enrichments using groundwater obtained from TAK-1, HRS, and YZ-50 (Fig. 4 and S6). In contrast, in the enrichments using groundwater obtained from all other sites, H_2_ and CO_2_ were initially generated within 48 h and accumulated, and then H_2_ decreased to below the detection limit. Following the disappearance of H_2_, CH_4_ production began to be observed. Incubations at temperatures that were 10°C higher than those of groundwater measured at ground level were suggested to exhibit a strong potential for CH_4_ production. In order to identify microbes in the enrichments suggested to actively produce biogas, archaeal and bacterial 16S rRNA gene clone libraries were constructed. Enrichments using groundwater from YZ-50, KOZ, and ART were used in the 16S rRNA gene analysis because the salinity of these groundwater samples had different signatures. The 16S rRNA gene analysis suggested that H_2_-utilizing methanogenic archaea and H_2_-producing fermentative bacteria were predominant in the enrichments, and that they belonged to the order *Methanobacteriales* and orders *Bacteroidales* and *Clostridiales*, respectively (4, 63, 64) (Fig. S7 and S8). The archaeal 16S rRNA gene was not amplified from the enrichment using groundwater obtained from YZ-50 by PCR after repeated attempts.

### Potential for microbial denitrification

We tested the potential for N_2_ production in deep aquifers associated with the accretionary prism. N_2_ production was recently discovered in freshwater sediments and was shown to be mediated by anaerobic CH_4_ oxidation coupled to denitrification (12, 42). In the present experiment, we used groundwater samples from US-2, ART, and IGH because large amounts of N_2_ and CH_4_ were detected in natural gas obtained from these sites. Consequently, N_2_ was rapidly produced in the enrichments using groundwater supplemented with nitrate or nitrite and both CH_4_ and YPG medium as electron donors (Fig. S9 and S10). A particularly strong potential for N_2_ production was observed in the enrichments incubated at the temperatures of groundwater measured at ground level. In contrast, N_2_ production was not observed in the enrichments amended with nitrate or nitrite and only CH_4_ as an electron donor.

In order to elucidate the phylogenetic positions of the members with enhanced N_2_ production induced by groundwater from the ART site, bacterial 16S rRNA genes derived from the enrichment were analyzed. The enrichment using groundwater from ART, which was supplemented with nitrate, CH_4_, and YPG medium and was indicated to have the highest N_2_ production rates, was used in the 16S rRNA gene analysis. The bacterial 16S rRNA genes revealed the dominance of the genus *Thauera* belonging to the order *Rhodocyclales* (Fig. S11). Species with validly published names in the genus *Thauera* have been isolated from various environments, and these species are reported to be capable of nitrate reduction and denitrification using organic matter as electron donors under anaerobic conditions (34).

## Discussion

**Fig. 5:**
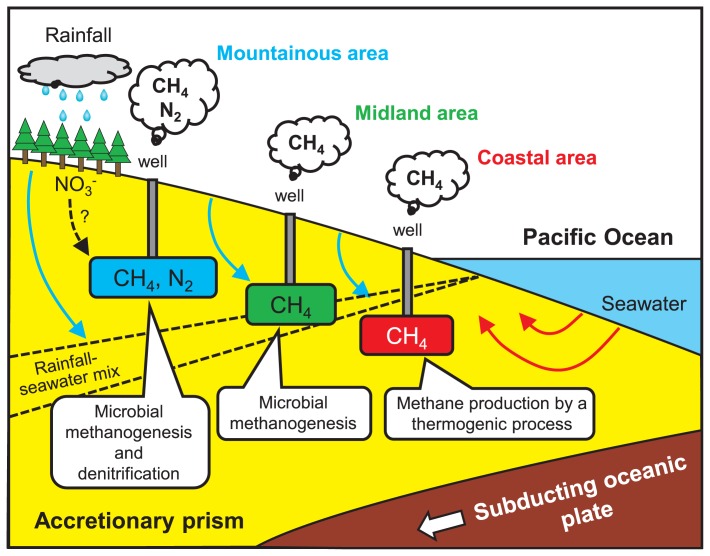
Schematic of a groundwater flow model and CH_4_ and N_2_ production processes in deep aquifers of the coastal area, midland area, and mountainous area associated with the accretionary prism in southwest Japan.

TAK-1, HRS, and YZ-50 are located in a coastal region at an altitude of 2 m, close to Suruga Bay within the Philippine Sea subducting plate (Fig. 1 and Table S1). Natural gas from the three sites contained a high concentration of CH_4_ (>97%). Our stable isotope analysis using the δ^13^C_CH4_ versus δ^13^C_∑CO2_ diagram showed that CH_4_ from the three sites is of thermogenic origin or is derived from both biogenic and thermogenic origins (Fig. 2). The enrichments using groundwater collected from the sites were indicated to have weak potential for CH_4_ production by microbial communities (Fig. 4 and S6). We simply estimated the geothermal gradient at each sampling site based on the well depth and temperature of groundwater at the outflow of the wells (Table S1). HRS and YZ-50 were suggested to have markedly higher geothermal gradients than those of the other sites. These high geothermal gradients may have been due to faults or fractures associated with previous earthquakes that took place in Suruga Bay, close to the Tokai subduction zone (3). On the other hand, the geothermal gradient at the TAK-1 site was not particularly high. In the well at the TAK-1 site, groundwater is pumped up using a gas lift system, in which natural gas obtained from the deep aquifer is separated from groundwater, cooled for dehydration, and injected into the deep aquifer using a gas compressor (Table S1). Thus, groundwater from the well at TAK-1 is considered to be cooled by mixing with injected gas, and the actual geothermal gradient is higher than that calculated in this study (33°C km^−1^). Our results based on the stable isotopic analysis, enrichments, and geothermal gradients suggest that CH_4_ is generated by the breakdown of organic compounds through a thermogenic process in the deep aquifers of TAK-1, HRS, and YZ-50 located in the coastal area associated with the accretionary prism (Fig. 5).

On the other hand, the concentrations of the larger hydrocarbons (*e.g.*, C_2_H_6_) were low in natural gas obtained from the three sites (see Table 1), which is inconsistent with CH_4_ production occurring through a thermogenic process (5). This result may have been due to non-methane hydrocarbons being stripped off during gas migration because larger hydrocarbons are more likely to be absorbed on sediment particles (47, 62).

Sites other than TAK-1, HRS, and YZ-50 are also located at an altitude of 24 m to 727 m in the midland or mountainous areas associated with the accretionary prism (Table S1). Natural gas collected from YWR, NKK, EIS, SMD, KOZ, and NGY contained high concentrations of CH_4_ (>96 vol.%), whereas that from KAW, US-2, ART, and IGH contained CH_4_ in the proportion of 49–83 vol.%. The stable carbon isotope ratios of CH_4_ and ∑CO_2_ from the ten sites ranged between −69.4‰ and −37.0‰ and between −9.10‰ and 19.0‰, respectively (Table S3). We noted the α_c_ value indicating offsets between δ^13^C_∑CO2_ and δ^13^C_CH4_ because microbial CH_4_ production in anaerobic subterranean environments is mainly mediated by H_2_/CO_2_-utilizing methanogenic archaea (29). α_c_ values varied from 1.04 to 1.08, except in the case of KAW, which suggests that CH_4_ obtained from sites in the midland and mountainous area is biogenic in origin via H_2_/CO_2_-utilizing methanogenesis or is a mixture of biogenic and thermogenic origins (Fig. 2).

The CARD-FISH analysis targeting archaeal 16S rRNA suggested that archaeal cells are included at a proportion of 5.5% to 14.1% of all cells in groundwater samples. Additionally, the NGS analysis revealed the dominance of H_2_-utilizing or acetate-utilizing methanogenic archaea in the archaeal communities. In order to measure the potential of CH_4_ production by methanogens, we performed anaerobic cultivation using groundwater amended with methanogenic substrates. However, the strong potential for CH_4_ production was only observed for the enrichments amended with H_2_/CO_2_ using groundwater collected from NKK, SMD, and NGY (Fig. S2 and S3). This may have been due to the growth inhibition of H_2_-utilizing methanogens caused by the pH change in enrichments supplemented with H_2_/CO_2_ or by the use of a high concentration of methanogenic substrates (45).

The NGS analysis targeting bacterial 16S rRNA genes showed the presence of fermentative bacteria belonging to the orders *Nitrospirales*, *Bacteroidales*, *Lactobacillales*, *Clostridiales*, and *Ignavibacteriales* in groundwater. Previous studies reported that fermentative bacteria belonging to these bacterial groups are able to degrade organic matter to H_2_ and CO_2_ or acetate under anaerobic environments (4, 18, 32, 38). In addition, some species belonging to the classes *Alphaproteobacteria*, *Betaproteobacteria*, and *Gammaproteobacteria* have been shown to possess the ability to grow by fermentation under anaerobic environments (22, 24, 59). Our results suggest that these bacteria grow by fermentation and degrade organic matter to H_2_ and CO_2_ or acetate in deep aquifers, which is supported by enrichments using groundwater amended with YPG medium and BES having a strong potential for H_2_ and CO_2_ production (Fig. S4 and S5).

The biodegradation of organic matter under anaerobic conditions is achieved by the cooperative catabolism of diverse bacteria and archaea. In particular, a syntrophic consortium between H_2_-producing fermentative bacteria and H_2_-utilizing methanogens is known to lead to the conversion of organic matter to CH_4_ in anaerobic environments (40, 45, 49). Therefore, we tested the potential for CH_4_ production by syntrophic biodegradation based on enrichments using groundwater amended with organic substrates. We observed rapid H_2_ production/consumption and significant CH_4_ production in these enrichments (Fig. 4 and S6). These dynamics of H_2_ and CH_4_ were clearly similar to those observed previously in syntrophic co-cultures of H_2_-producing fermentative bacteria and H_2_-utilizing methanogens (19, 29). Additionally, 16S rRNA gene analyses showed that H_2_-producing fermentative bacteria and H_2_-utilizing methanogenic archaea grew in these enrichments (Fig. S7 and S8). The results based on the stable isotopic analysis and microbiological assays strongly suggest that a syntrophic consortium of H_2_-producing fermentative bacteria and H_2_-utilizing methanogenic archaea contributes to CH_4_ production in the deep aquifers in sites situated in midland and mountainous areas (Fig. 5). In addition, it currently remains unclear which chemical components of organic matter are actually used in this fermentation and CH_4_ production. Future studies may identify these components.

The natural gas samples obtained from KAW, US-2, ART, and IGH contained large amounts of N_2_ (15–50 vol.%). These sites are located at relatively high altitudes in the mountainous area associated with the accretionary prism. N_2_ in natural gas may have been produced through microbial denitrification; nitrate is reduced to N_2_ via the intermediate steps for nitrite, nitric oxide, and nitrous oxide (23). Nitrate was below the detection limit in groundwater obtained in this study, possibly due to a high level of nitrate consumption via the denitrification process. We herein measured N_2_ and Ar concentrations in natural gas samples and calculated the N_2_/Ar ratio. The amount of N_2_ may be altered by biochemical reactions, whereas that of the noble gas Ar is expected to remain constant. If nitrate (or nitrite) is transformed into N_2_ by microbial denitrification, the N_2_/Ar ratio will be changed. The results of the gas analysis demonstrated that the N_2_/Ar ratio ranged between 41 and 203 in natural gas collected from the four sites (Table 1). Air has an N_2_/Ar ratio of 84, whereas air-saturated water has an N_2_/Ar ratio of approximately 40 (9, 15). The N_2_/Ar ratios of natural gas derived from the four sites were higher than that of air-saturated water.

N_2_ production was not observed in the enrichments amended with nitrate or nitrite and only CH_4_ as an electron donor using groundwater (Fig. S9 and S10). Therefore, denitrification coupled with anaerobic CH_4_ oxidation may not occur in deep aquifers. On the other hand, the enrichments amended with nitrate or nitrite and organic matter and the 16S rRNA gene analysis suggest that denitrifying bacteria using organic matter as an electron donor exhibit a strong potential for N_2_ production (see Fig. S9, S10, and S11). These results suggest that N_2_ produced by microbial denitrification using organic matter is present in deep aquifers in sites located in the mountainous area (Fig. 5).

These denitrifying bacteria may compete with the CH_4_-producing syntrophic consortium for organic matter in accretionary prism sediments. The competitiveness of denitrifying bacteria may be measured based on the amount of nitrate present in the deep aquifer containing organic compounds, but only limited amounts of nitrate. Previous studies reported that excess amounts of nitrate are present in surface environments such as forest soil, and that it is lost via water movement (17, 48). Therefore, groundwater may deliver nitrate to deep aquifers associated with the accretionary prism. Although the KAW site was an exception, the salinity of groundwater obtained from US-2, ART, and IGH was significantly lower than that at the other sites (*P*<0.005 by the Student’s *t*-test). This result suggests that deep aquifers in the three sites were strongly affected by rainfall, and rainfall may supply them with nitrate, which is consistent with our detection of a significant amount of N_2_ only from natural gas derived from the deep aquifer in the mountainous area.

Although natural gas obtained from KAW contained 15 vol.% of N_2_, groundwater was indicated to have high salinity (2,450 mS m^−1^). It has been proposed that a fault or fracture zone may be present at the KAW site (37). Therefore, the deep aquifer in the KAW site may be affected by rainfall and high-salinity groundwater that rises from the deep subterranean environment through fault or fracture zones. The clarification of groundwater flow in the subterranean environment associated with the accretionary prism will be an important topic for future study.

## Conclusion

The chemical, stable isotopic, and microbiological data obtained in this study demonstrate regional variations in CH_4_ and N_2_ production processes. CH_4_ is produced by a thermogenic process, particularly in the deep aquifer in the coastal area associated with the accretionary prism, and H_2_-producing fermentative bacteria and H_2_-utilizing methanogens contribute to the significant production of CH_4_ in midland and mountainous areas. In addition, the production of N_2_ in the deep aquifer in the mountainous area is mediated by denitrifying bacteria that use organic matter as an electron donor. Overall, these results lead us to the conclusion that dynamic groundwater flow and the ongoing biodegradation of organic matter in ancient sediments contribute to CH_4_ and N_2_ reserves in deep aquifers associated with the accretionary prism in southwest Japan.

## Supplementary Information



## Figures and Tables

**Table 1:** Physical and chemical parameters and microbial cell density of groundwater and components of natural gas.

Site code	Groundwater					Natural gas					
	
	Temp. (°C)	pH	ORP (mV)	EC (mS m^−1^)	Microbial cells (cells mL^−1^)	N_2_ (vol.%)	Ar (vol.%)	CH_4_ (vol.%)	C_2_H_6_ (vol.%)	C_3_H_8_ (vol.%)	N_2_/Ar ratio
TAK-1	49.3	8.5	−175	3,090	3.0×10^3^	2.86	0.18	97.0	0.028	n.d.	16
HRS	40.8	8.4	−183	2,590	7.7×10^5^	1.18	0.19	98.6	0.016	n.d.	6
KAW	48.7	7.6	−196	2,450	4.5×10^3^	15.8	0.25	83.8	0.061	n.d.	63
YZ-50	41.0	7.8	−114	2,050	2.7×10^4^	1.98	0.10	97.9	0.018	n.d.	20
YWR	24.2	8.0	−226	1,747	1.7×10^4^	1.19	0.06	98.7	0.009	n.d.	20
NKK	40.6	8.2	−178	1,619	5.6×10^4^	2.37	0.07	97.5	0.152	n.d.	34
EIS	28.6	8.4	−312	586	4.0×10^4^	2.46	0.08	97.4	0.063	n.d.	31
SMD	39.0	8.2	−270	559	1.4×10^4^	0.79	0.06	96.9	2.248	n.d.	13
KOZ	30.0	8.3	−297	546	3.5×10^4^	3.21	0.07	96.7	0.018	n.d.	46
NGY	26.7	9.3	−320	479	1.1×10^5^	3.35	0.09	96.5	0.058	n.d.	37
US-2	32.8	8.9	−255	170	3.8×10^4^	23.5	0.58	76.0	0.009	n.d.	41
ART	35.1	8.8	−257	147	2.5×10^4^	50.2	0.25	49.5	0.015	n.d.	203
IGH	31.2	8.7	−265	110	1.2×10^4^	36.4	0.66	63.0	0.004	n.d.	55

Abbreviations: ORP, oxidation-reduction potential; EC, electric conductivity; n.d. not detected.
