# Supplementary material for: Regional Variation of CH4 and N2 Production Processes in the Deep Aquifers of an Accretionary Prism
Source: Microbes Environ. 2016 Sep 3;31(3):329–38. doi: 10.1264/jsme2.ME16091 (PMC5017811; doi:10.1264/jsme2.ME16091)
Supplement: Supplementary file 1 [file 31_329_s1.pdf]

Table S1. Location and structure of wells for sampling and estimated geothermal gradient.

Site name	Site code	Latitude	Longitude	Altitude (m)	Well depth (m)	Strainer depth (m)	Water pumping method	Geothermal gradient ($^{o}C\ km^{-1}$)
Takakusa No.1	TAK-1	34°52'32.31"N	138°19'25.30"E	2	1,500	1,000-1,500	GLS	33
Houraiso	HRS	34°52'12.42"N	138°19'17.60"E	2	150	150	NWP	272
Kawane	KAW	34°57'35.33"N	138°05'22.61"E	153	1,148	1,048-1,148	NWP	42
Yaizu No. 50	YZ-50	34°52'28.12"N	138°19'17.86"E	2	170	129-170	WP	241
Ensyu Yawaragi	YWR	34°43'44.54"N	137°55'29.61"E	24	1,500	no data	WP	16
Narakoko	NKK	34°52'30.81"N	138°00'55.42"E	176	1,500	1,300-1,500	WP	27
Eisei Kakegawa	EIS	34°49'06.82"N	138°01'12.44"E	45	1,000	no data	NWP	29
Itawari	SMD	34°52'18.50"N	138°09'17.64"E	148	1,489	1,189-1,481	WP	26
Kozeto	KOZ	34°59'11.23"N	138°17'54.18"E	56	910	800-910	NWP	33
Nishigaya	NGY	35°00'53.76"N	138°20'57.55"E	54	1,500	1,191-1,480	WP	18
Umegashima Shinden No. 2	US-2	35°16'58.10"N	138°20'19.66"E	727	1,000	700-1,000	WP	33
Aratama	ART	34°50'37.72"N	137°45'58.38"E	112	1,500	777-1,500	WP	23
Iwata Grand Hotel	IGH	34°44'09.26"N	137°52'47.08"E	43	1,500	1,400-1,500	WP	21

Abbreviation: GLS, gas lift system; NWP, natural water pressure; WP, water pump.

Geothermal gradient was simply estimated based on well depth and temperature of groundwater measured at the outflow of the wells.

Table S2. The chemical characteristics of groundwater samples.

Site code	Na^{+} (mg L^{-1})	Ca^{2+} (mg L^{-1})	Mg^{+} (mg L^{-1})	K^{+} (mg L^{-1})	Fe^{2+} (mg L^{-1})	Fe^{3+} (mg L^{-1})	Cl^{-} (mg L^{-1})	Br^{-} (mg L^{-1})	I^{-} (mg L^{-1})	F^{-} (mg L^{-1})	PO_4^{2-} (mg L^{-1})	NO_2^{-} (mg L^{-1})	NO_3^{-} (mg L^{-1})	NH_4^{+} (mg L^{-1})	SO_4^{2-} (mg L^{-1})	S^{2-} (mg L^{-1})	HCO_3^{-} (mg L^{-1})	Acetate (mg L^{-1})	Formate (mg L^{-1})	DOC (mg L^{-1})
TAK-1	3,100	2,200	n.d.	25	1.6	n.d.	9,500	33	6.0	n.d.	n.d.	n.d.	n.d.	15	0.1	n.d.	9.6	n.d.	n.d.	0.4
HRS	2,600	2,300	n.d.	20	n.d.	n.d.	7,400	27	5.0	n.d.	n.d.	n.d.	n.d.	6.0	0.1	n.d.	6.0	n.d.	n.d.	0.5
KAW	4,000	47	10	40	0.5	n.d.	6,200	13	4.0	3.9	n.d.	n.d.	n.d.	9.0	0.2	n.d.	330	n.d.	n.d.	0.3
YZ-50	2,100	2,100	3.0	17	0.1	n.d.	5,100	18	5.0	n.d.	n.d.	n.d.	n.d.	15	0.1	n.d.	27	n.d.	n.d.	n.d.
YWR	3,100	53	36	29	1.5	n.d.	4,100	21	11	n.d.	n.d.	n.d.	n.d.	9.6	n.d.	n.d.	780	n.d.	n.d.	50
NKK	3,000	22	7.0	27	0.1	0.3	3,800	9.0	n.d.	2.4	n.d.	n.d.	n.d.	7.8	0.1	n.d.	1,300	n.d.	n.d.	0.5
EIS	960	24	2.0	4.6	0.9	n.d.	1,200	3.8	2.0	12	n.d.	n.d.	n.d.	2.4	0.3	n.d.	110	n.d.	n.d.	0.6
SMD	850	7.5	n.d.	7.1	0.3	n.d.	1,100	0.5	3.0	6.9	n.d.	n.d.	n.d.	3.9	0.8	n.d.	581	n.d.	n.d.	0.8
KOZ	900	23	7.0	59	0.3	n.d.	800	0.1	1.0	14	n.d.	n.d.	n.d.	3.7	0.3	n.d.	1,100	n.d.	n.d.	0.6
NGY	760	1.6	n.d.	8.1	2.3	n.d.	880	3.0	4.0	9.9	n.d.	n.d.	0.1	5.0	0.7	n.d.	400	n.d.	n.d.	2.5
US-2	370	0.9	n.d.	2.8	n.d.	n.d.	2.7	n.d.	2.0	6.9	n.d.	n.d.	n.d.	2.5	0.6	n.d.	750	n.d.	n.d.	n.d.
ART	230	0.6	n.d.	3.0	1.4	n.d.	30	0.1	n.d.	12	n.d.	n.d.	n.d.	2.7	0.6	n.d.	642	n.d.	n.d.	n.d.
IGH	190	5.0	2.0	3.0	1.2	n.d.	130	0.4	n.d.	0.9	0.6	n.d.	n.d.	0.6	n.d.	n.d.	14	n.d.	n.d.	14

Abbreviation: DOC, dissolved organic carbon; n.d., not detected.

Table S3. Stable carbon isotope ratio of CH_4 in natural gas and dissolved inorganic carbon (ΣCO_2) in groundwater.

Site code	$\delta^{13}C_{CH4}$ (‰)	$\delta^{13}C_{\Sigma CO2}$ (‰)	α_c
TAK-1	−34.6	−8.76	1.027
HRS	−33.5	−9.76	1.025
KAW	−37.0	−3.27	1.035
YZ-50	−34.5	10.0	1.046
YWR	−69.4	1.53	1.076
NKK	−42.1	7.96	1.052
EIS	−48.4	18.4	1.070
SMD	−39.2	19.0	1.061
KOZ	−38.4	10.1	1.050
NGY	−39.3	13.3	1.055
US-2	−57.5	−1.26	1.060
ART	−52.2	−5.65	1.049
IGH	−65.4	−9.10	1.060

Abbreviation: $\alpha_c = (\delta^{13}C_{\Sigma CO2} + 10^3)/(\delta^{13}C_{CH4} + 10^3)$.

Table S4. Abundance of archaeal and bacterial cells in groundwater samples detected by CARD-FISH analysis.

Site code	Archaeal cells (%)	Bacterial cells (%)	Bacteria/Archaea ratio
KAW	11.1	13.3	1.2
YZ-50	68.0	7.1	0.1
NKK	5.5	7.1	1.3
EIS	14.1	32.2	2.3
SMD	7.0	7.9	1.1
KOZ	12.2	8.7	0.7
US-2	8.0	45.2	5.7
ART	12.0	12.6	1.1

The abundance of archaeal and bacterial cells were determined by the count ratio of FISH-positive cells to the SYBR Green I stained cells.

Table S5. Number of prokaryotic 16S rRNA gene sequences derived from groundwater and statistical estimators.

Site code	Total of sequences	No. of OTUs	Coverage (%)	Chao 1	Shannon
KAW	33,274	652	99.0	1,849	6.25
YZ-50	20,395	295	99.2	566	2.30
NKK	15,138	414	99.2	559	5.06
EIS	9,249	312	98.8	494	5.15
SMD	8,958	196	99.3	289	4.15
KOZ	16,257	459	99.4	578	6.14
US-2	7,787	128	99.3	217	1.18
ART	19,405	787	98.4	1,212	6.16

Abbreviations: OTU, operational taxonomic unit.

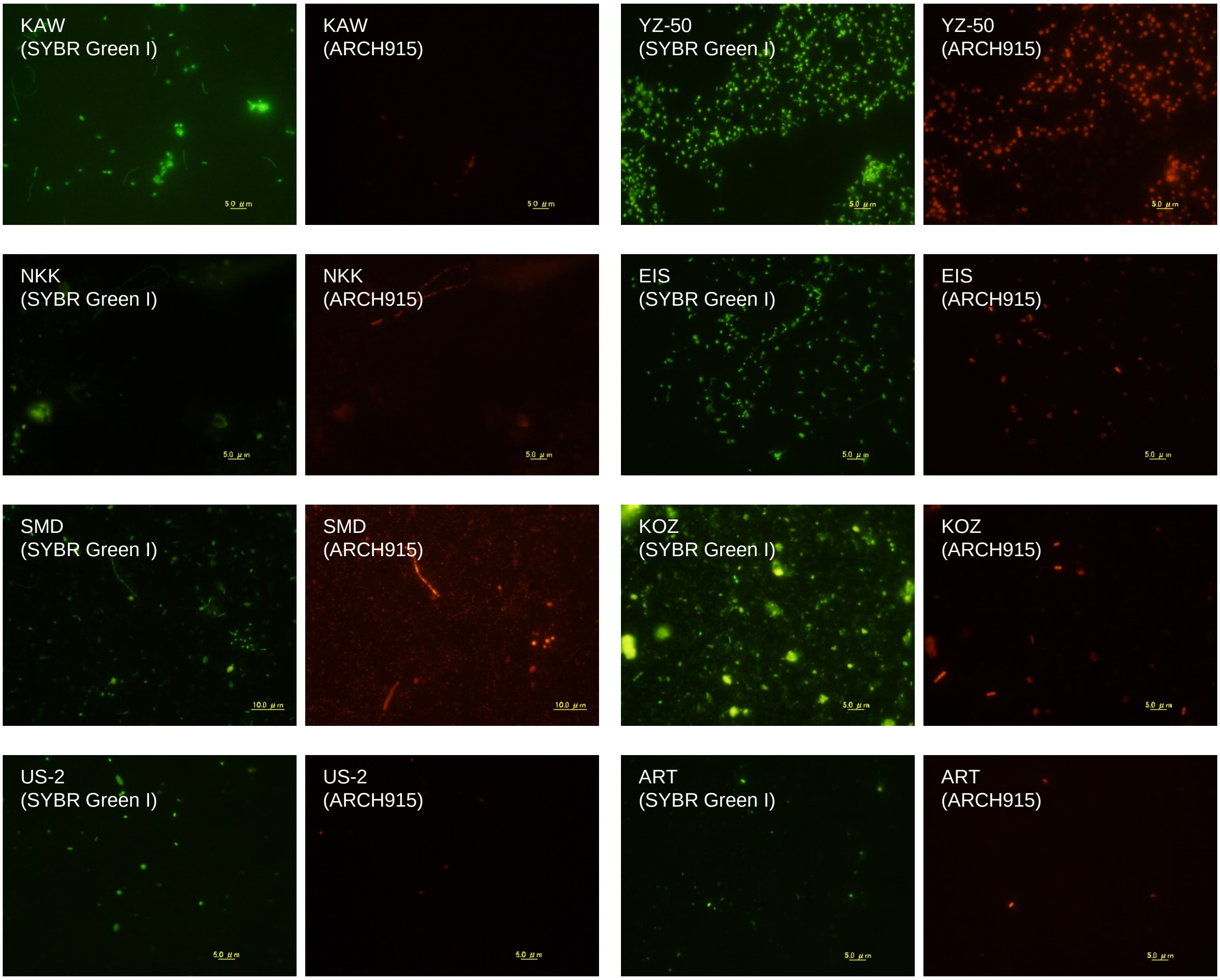

Fig. S1 CARD-FISH photomicrographs of microbial cells in groundwater samples. Each double panel shows identical fields and depicts microbial cells counterstained with SYBR Green I in green (left) and archaeal cells hybridized with *Archaea*-specific ARCH915 probe in red (right). Yellow signals are autofluorescence of clay particles.

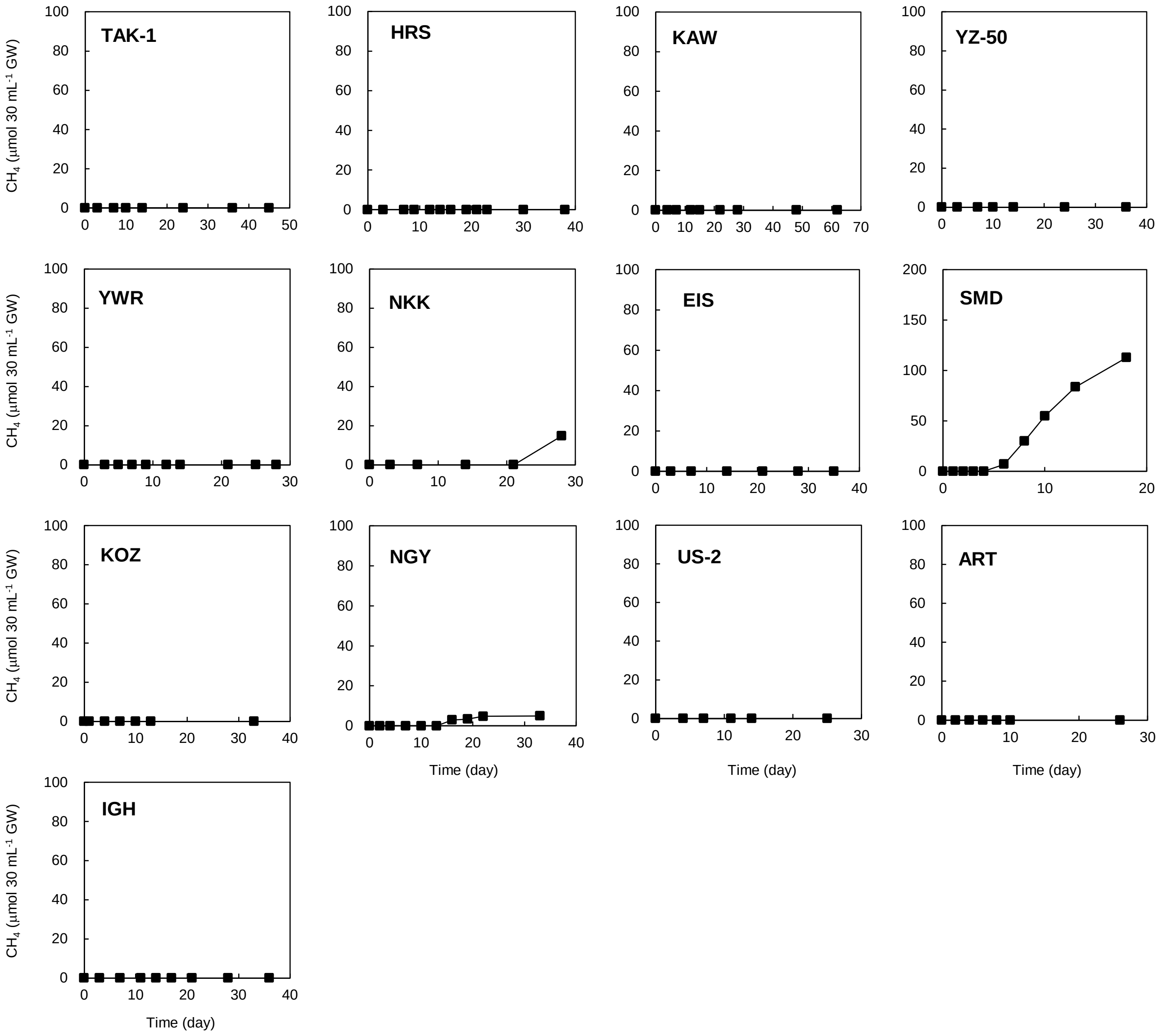

Fig. S2. CH_4 production from the groundwater samples amended with H_2/CO_2 incubated at each temperature of groundwater sample that was measured at the outflow of the well. Date points were obtained from the measurement of cumulative CH_4 (■) in the gas phase of the culture bottles.

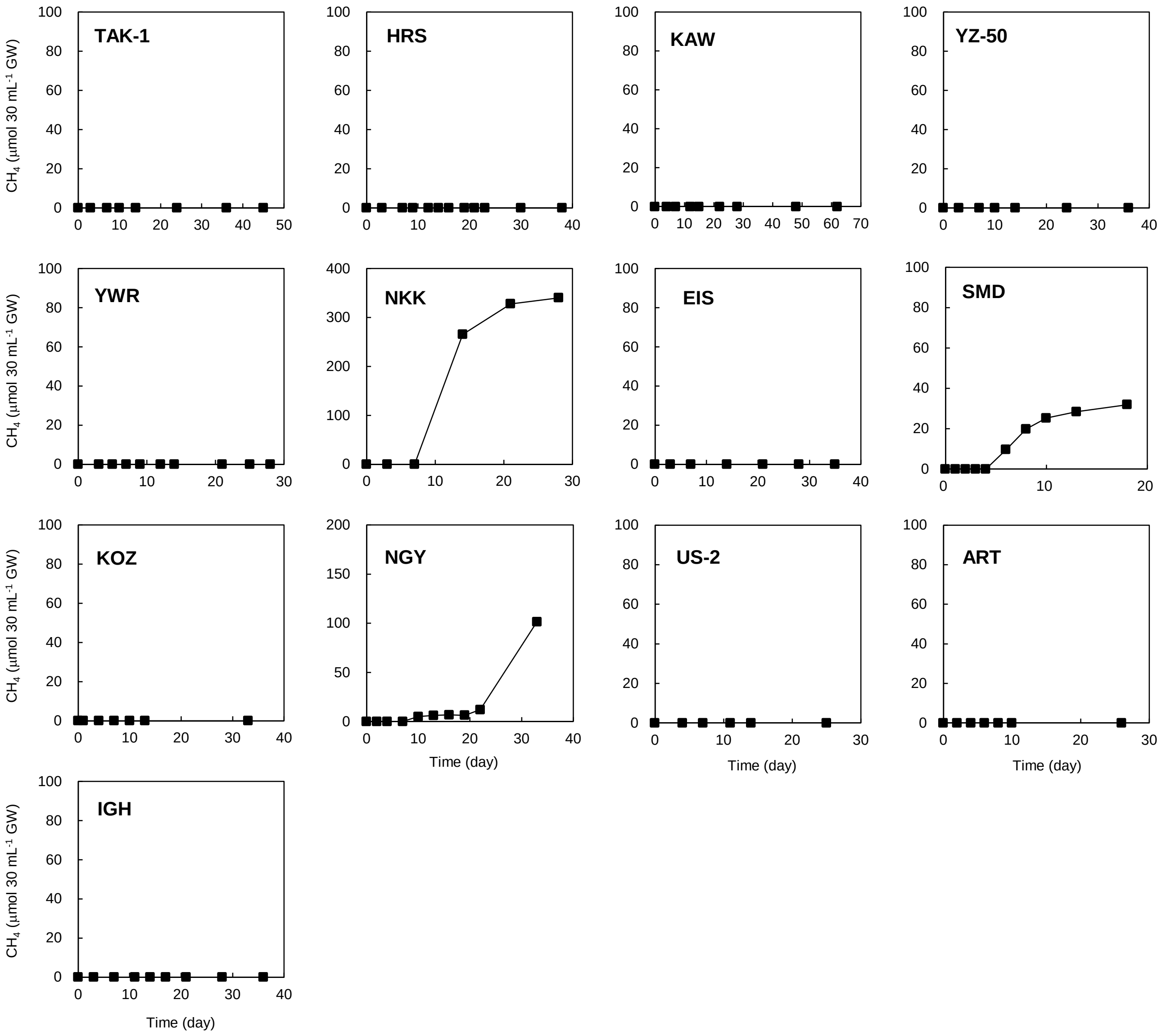

Fig. S3. CH_4 production from the groundwater samples amended with H_2/CO_2 incubated at temperatures 10ºC higher than those of groundwater samples that were measured at the outflow of the deep wells. Date points were obtained from the measurement of cumulative CH_4 (■) in the gas phase of the culture bottles.

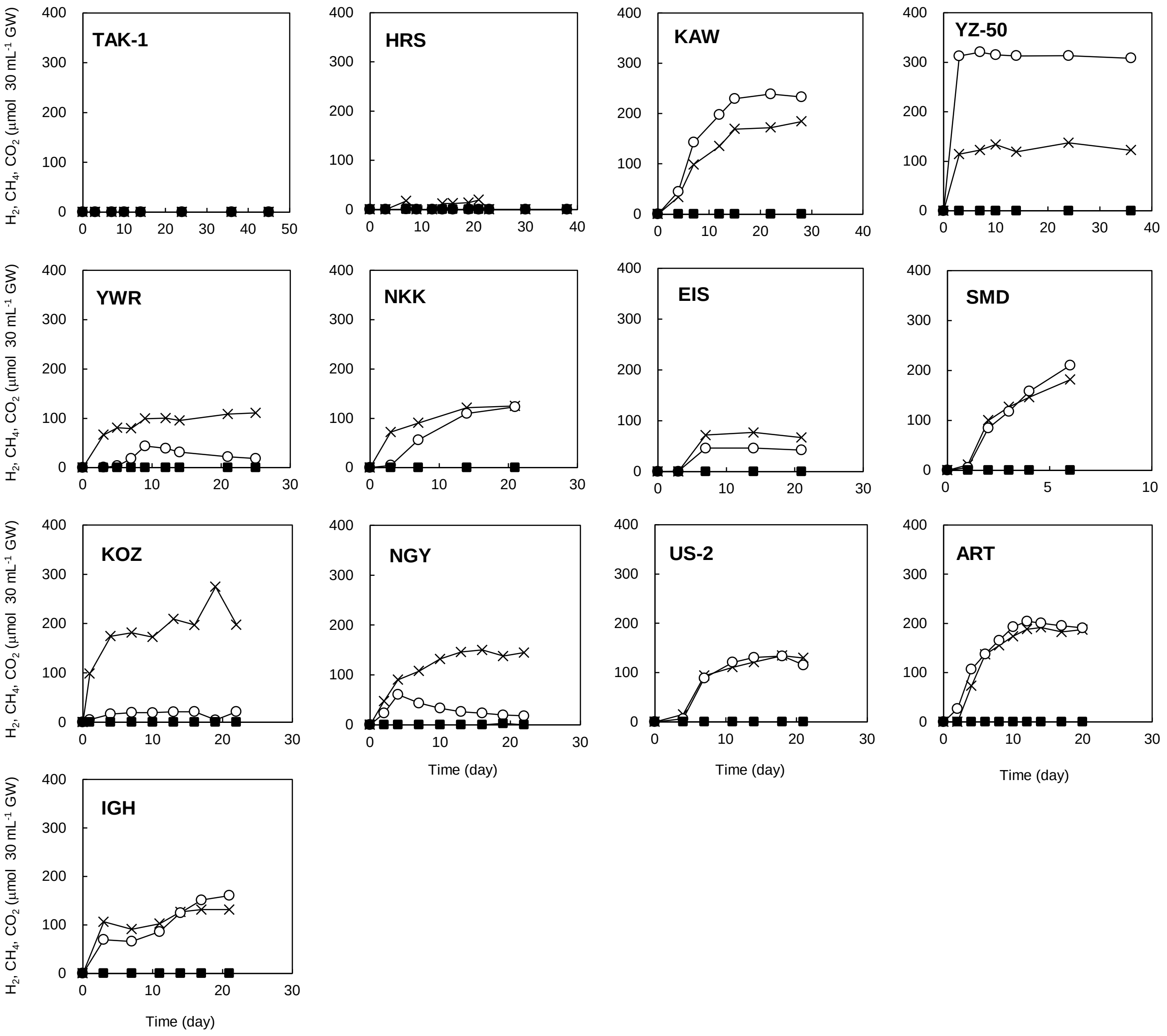

Fig. S4. Biogas production from the groundwater samples amended with YPG medium and 2-bromoethanesulfonate, an inhibitor of methanogens, incubated at each temperature of groundwater sample that was measured at the outflow of the deep well. Data points were obtained from the measurement of cumulative H_2 (○), CH_4 (■) and CO_2 (×) in the gas phase of the culture bottles.

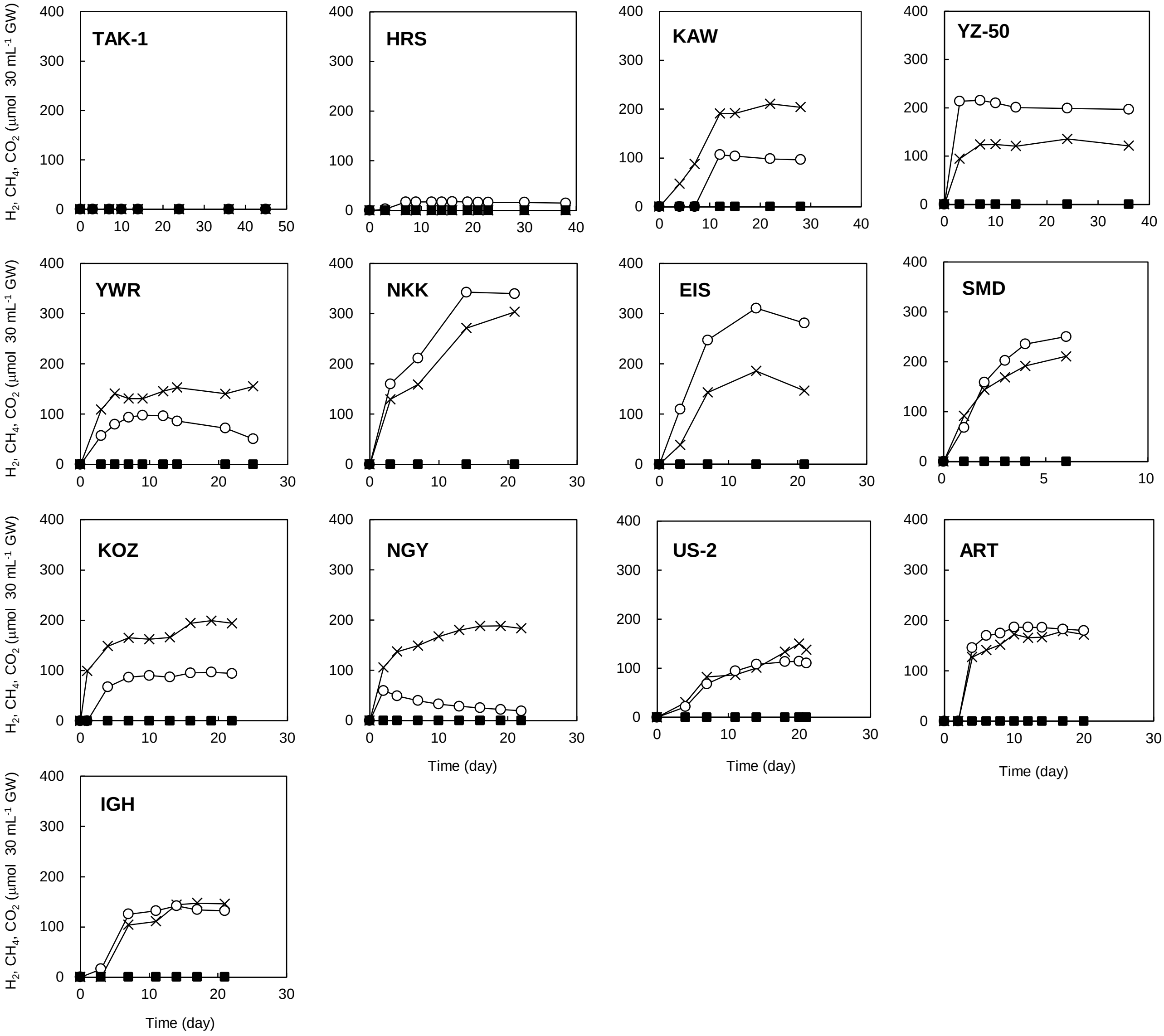

Fig. S5. Biogas production from the groundwater samples amended with YPG medium and 2-bromoethanesulfonate, an inhibitor of methanogens, incubated at temperatures 10ºC higher than those of groundwater samples that were measured at the outflow of the deep wells. Data points were obtained from the measurement of cumulative H_2 (○), CH_4 (■) and CO_2 (×) in the gas phase of the culture bottles.

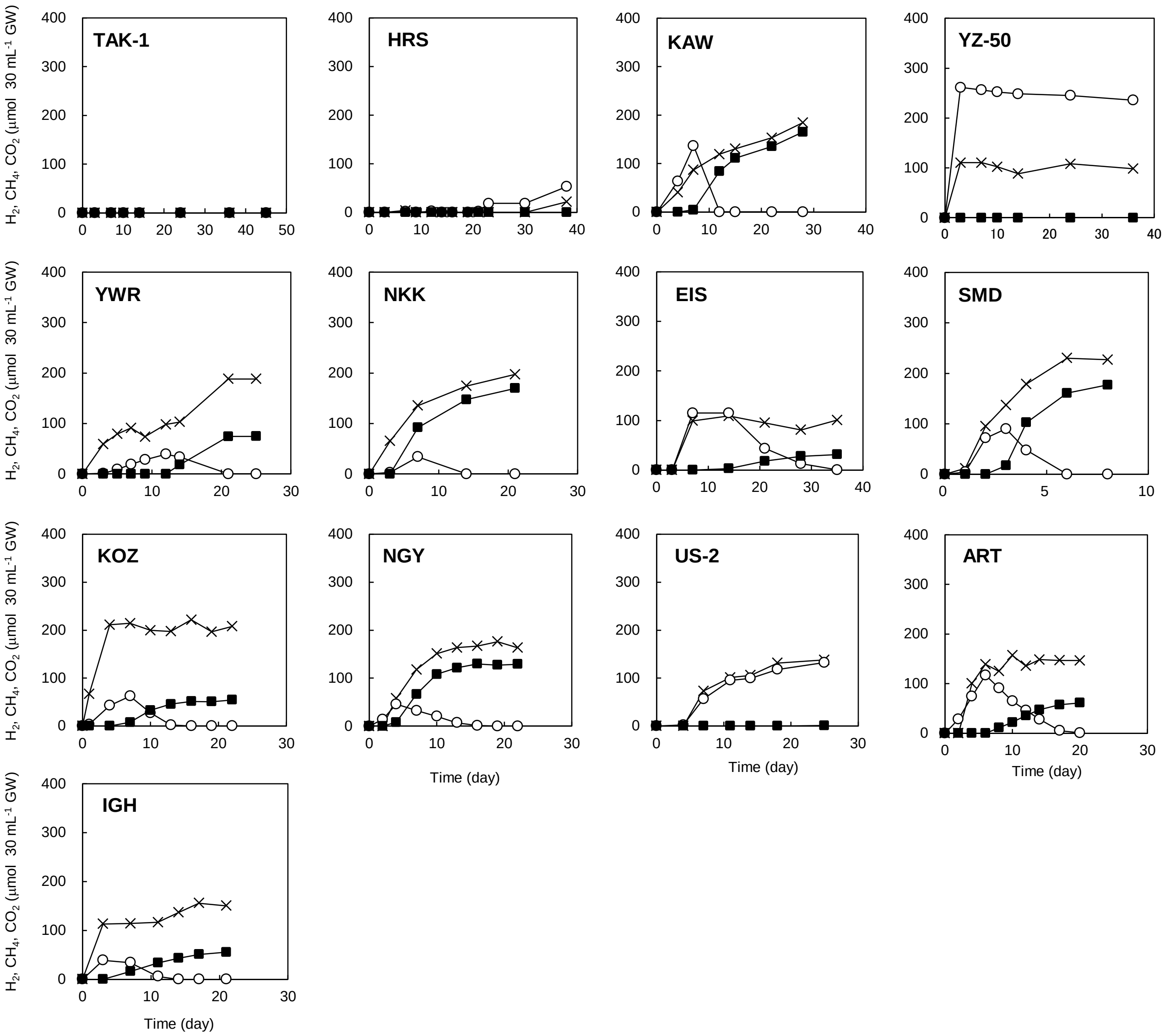

Fig. S6. Biogas production from the groundwater samples amended with YPG medium incubated at each temperature of groundwater sample that was measured at the outflow of the deep well. Data points were obtained from the measurement of cumulative H_2 (○), CH_4 (■) and CO_2 (×) in the gas phase of the culture bottles.

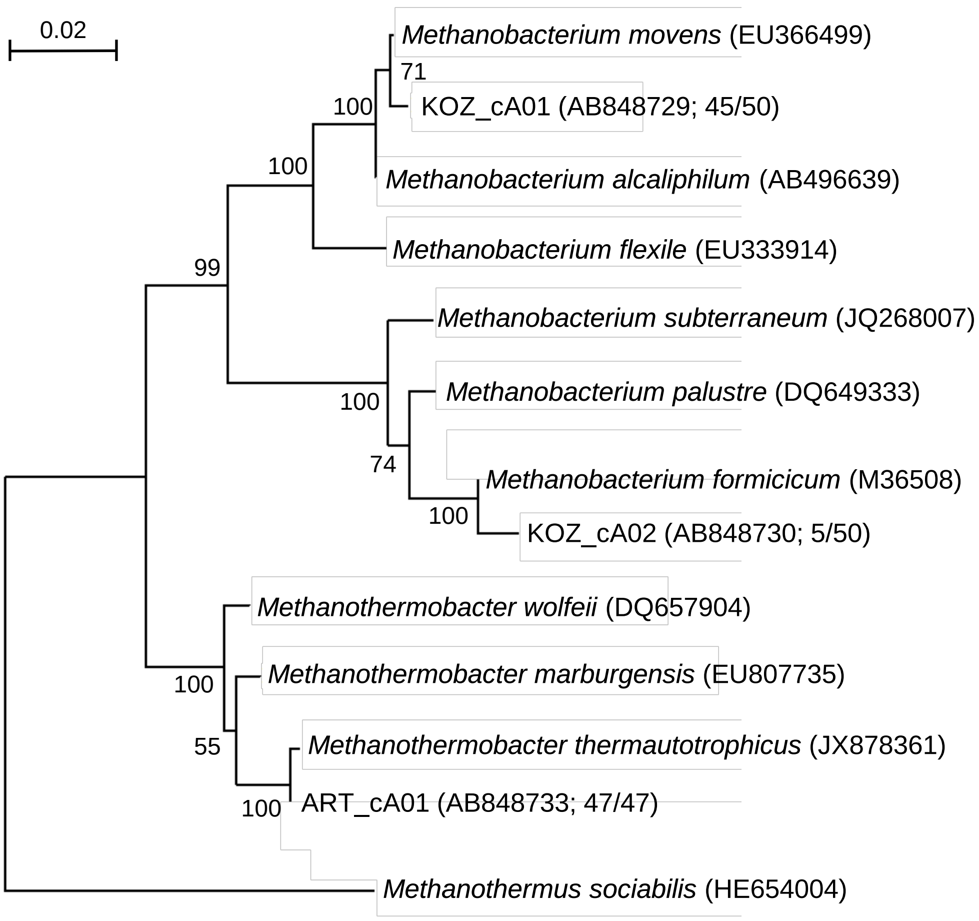

Fig. S7. Neighbor-joining tree of the archaeal 16S rRNA gene sequences derived from the enrichments using YPG medium-amended groundwater for CH_4 production. Three OTUs obtained in this study are shown with their relatives of established species whose sequences were retrieved from the public database. Accession numbers and the number of clones in each clone library are shown in parentheses. Bootstrap values determined from 1,000 iterations are indicated at branching points. The sequence of *Methanothermus sociabilis* (HE654004) was used as the outgroup to root the tree. Scale bar represents 2 substitution per 100 nucleotides.

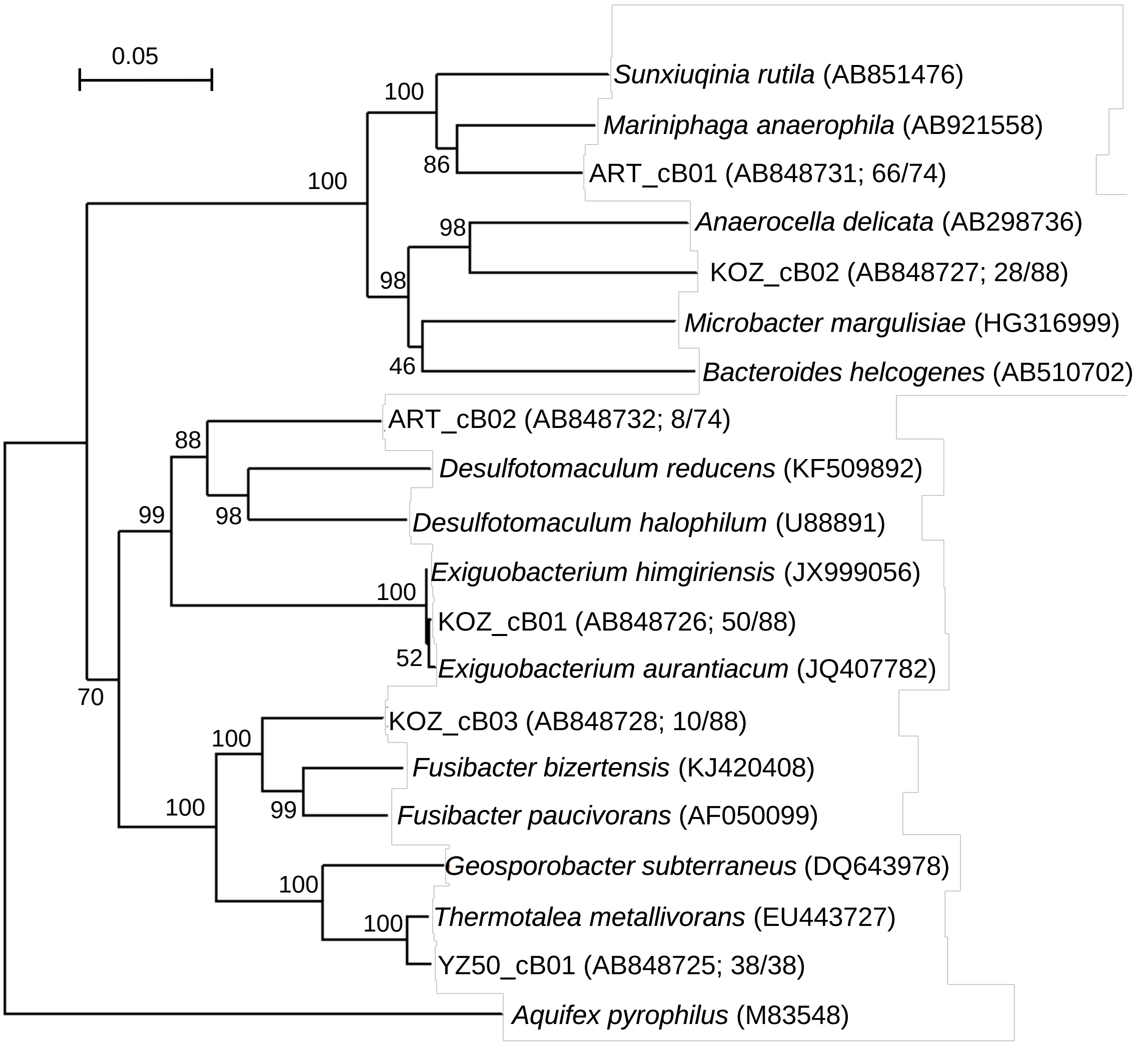

Fig. S8. Neighbor-joining tree of the bacterial 16S rRNA gene sequences derived from the enrichments using YPG medium-amended groundwater for CH_4 production. 6 OTUs obtained in this study are shown with their relatives of established species whose sequences were retrieved from the public database. Accession numbers and the number of clones in each clone library are shown in parentheses. Bootstrap values determined from 1,000 iterations are indicated at branching points. The sequence of *Aquifex pyrophilus* (MB83548) was used as the outgroup to root the tree. Scale bar represents 5 substitution per 100 nucleotides.

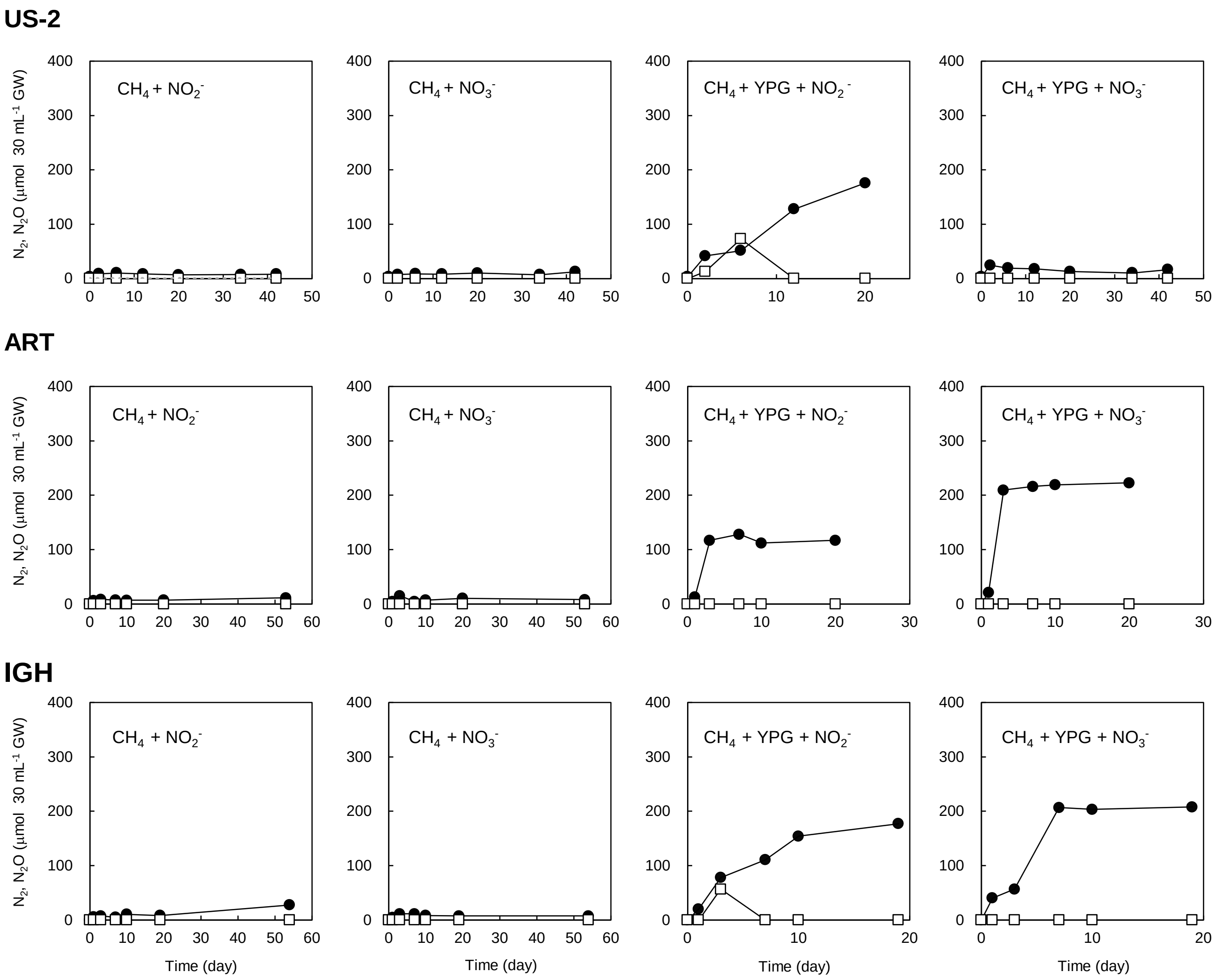

Fig. S9. N_2 and N_2O productions from the groundwater samples amended with CH_4 (and YPG medium) and nitrite (or nitrate) incubated at each temperature of groundwater sample that was measured at the outflow of the deep well. Data points were obtained from the measurement of cumulative N_2 (●) and N_2O (□) in the gas phase of the culture bottles.

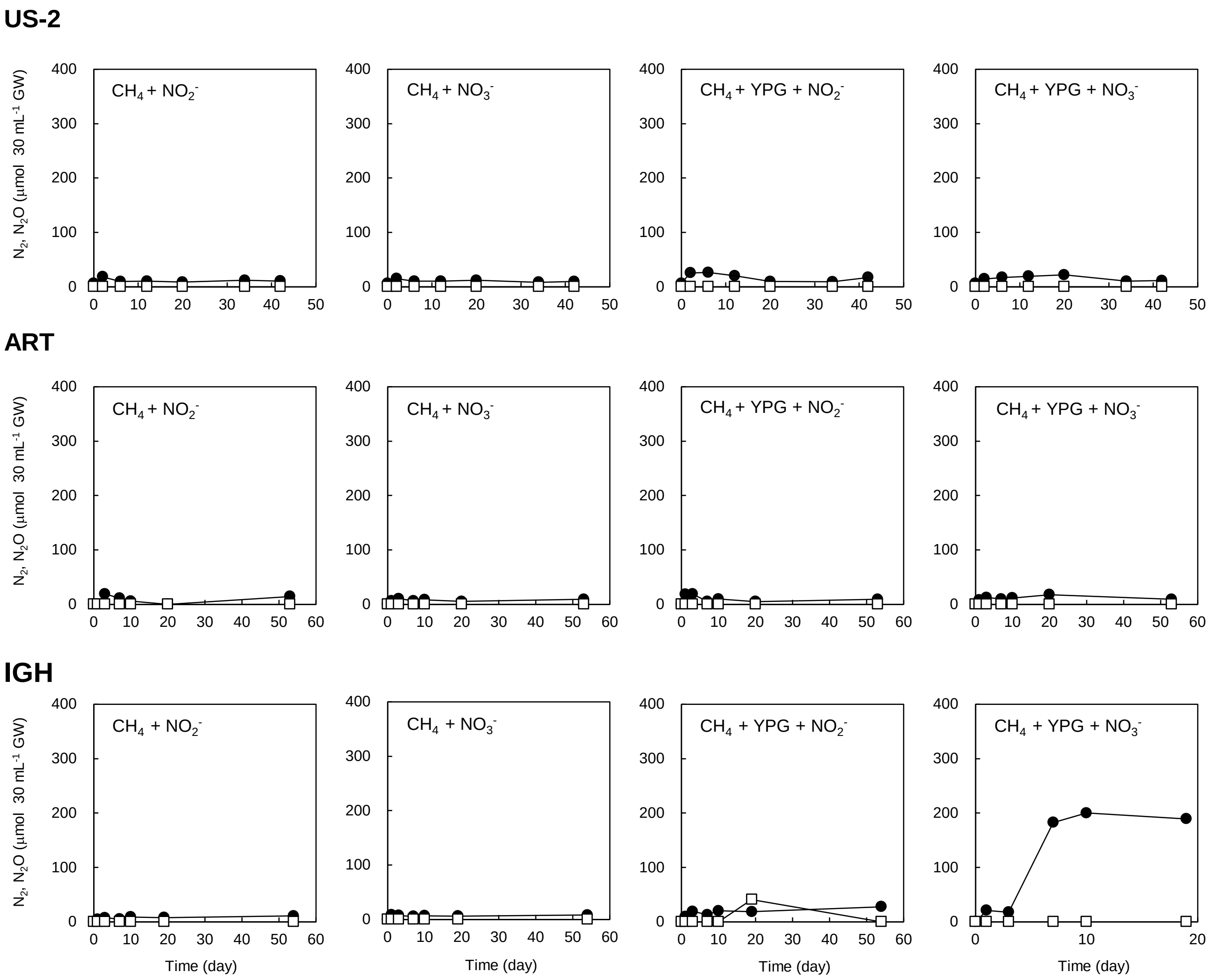

Fig. S10. N_2 and N_2O productions from the groundwater samples amended with CH_4 (and YPG medium) and nitrite (or nitrate) incubated at temperatures 10ºC higher than those of groundwater samples that were measured at the outflow of the deep wells. Data points were obtained from the measurement of cumulative N_2 (●) and N_2O (□) in the gas phase of the culture bottles.

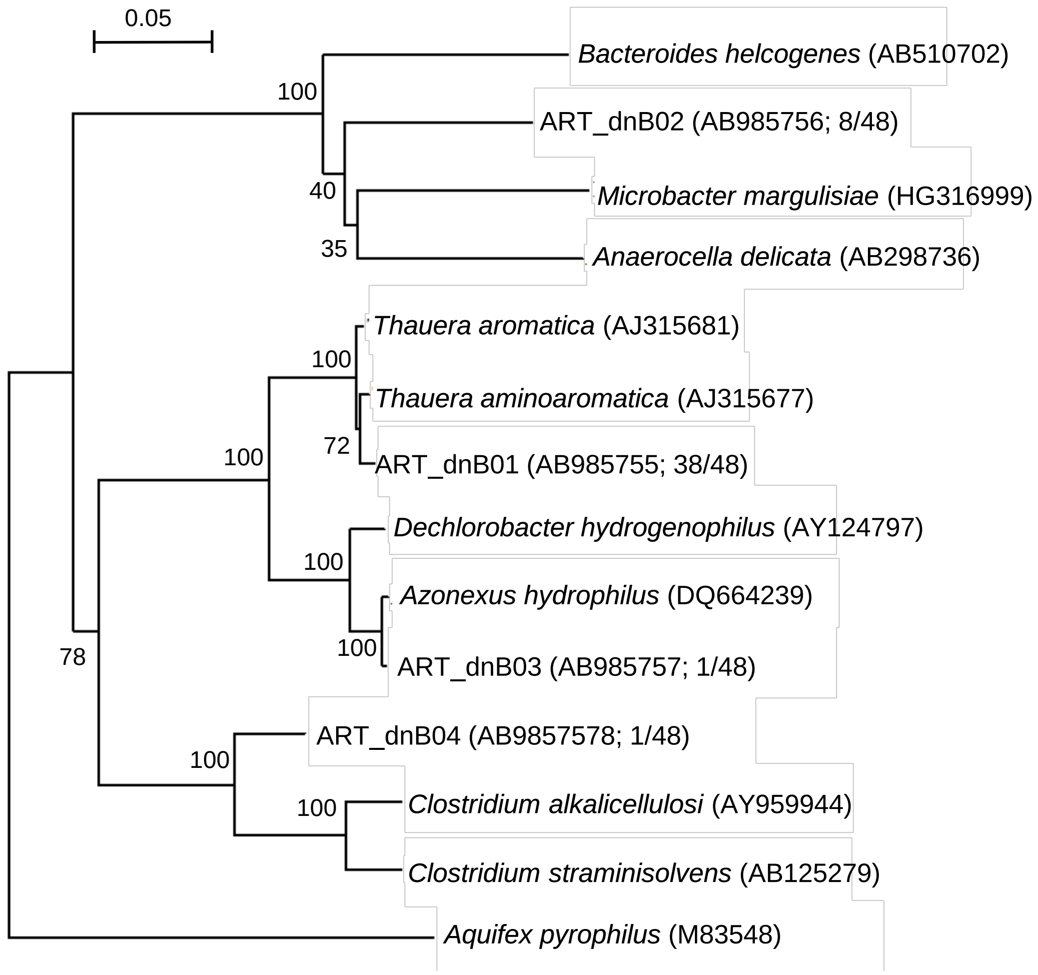

Fig. S11. Neighbor-joining tree of the bacterial 16S rRNA gene sequences derived from the enrichments using CH_4, YPG medium, and nitrate-amended groundwater for N_2 production. 4 OTUs obtained in this study are shown with their relatives of established species whose sequences were retrieved from the public database. Accession numbers and the number of clones in clone library are shown in parentheses. Bootstrap values determined from 1,000 iterations are indicated at branching points. The sequence of *Aquifex pyrophilus* (MB83548) was used as the outgroup to root the tree. Scale bar represents 5 substitution per 100 nucleotides.
